# Cytogenetic microarray in prenatal and postnatal diagnosis

**DOI:** 10.1186/1755-8166-7-S1-I32

**Published:** 2014-01-21

**Authors:** Shubha Phadke

**Affiliations:** 1 Department of Medical Genetics, SGPGIMS, Lucknow, India

## 

Due to high resolution cytogenetic microarray (CMA) has replaced traditional karyotyping for evaluation of individuals with intellectual disability, autism and congenital malformations. The diagnostic yield of CMA is 10 to 12 % and is more than any other investigation for evaluation of developmental disabilities. Due to its high resolution CMA is also being used as a prenatal test for chromosomal anomalies. The diagnostic yield is about 3% whatever may be the indication. The yield is higher in cases with fetal anomalies. The main concern with CMA is its ability detection of copy number variations of unknown significance. This is a major problem in prenatal diagnosis and is a challenge for the counselor and dilemma for the family in concern. With accumulation of more and more data of pathological and polymorphic variations in genome the variations of unknown significance will decrease. CMA is also useful in delineating abnormalities picked up by karyotyping. Some cases with double segment rearrangement may point towards familial chromosomal rearrangement and hence, CMA is indicated in familial cases with developmental disabilities and birth defects.

Cost and availability of clinical cytogeneticists for appropriate interpretation of CMA results are important concerns for wider application of the technique.

